# *Bifidobacterium bifidum* OLB6378 Simultaneously Enhances Systemic and Mucosal Humoral Immunity in Low Birth Weight Infants: A Non-Randomized Study

**DOI:** 10.3390/nu9030195

**Published:** 2017-02-26

**Authors:** Katsunori Tanaka, Takamitsu Tsukahara, Takahide Yanagi, Sayuri Nakahara, Ouki Furukawa, Hidemi Tsutsui, Shigeki Koshida

**Affiliations:** 1 Department of Pediatrics, Shiga University of Medical Science, Seta Tsukinowa-cho, Otsu, Shiga 520-2192, Japan; tanakatsu0124@gmail.com (K.T.); tyanagi@belle.shiga-med.ac.jp (T.Y.); sayuri10@belle.shiga-med.ac.jp (S.N.); ouki@belle.shiga-med.ac.jp (O.F.); hidemizura@yahoo.co.jp (H.T.); 2 Laboratory of Animal Science, Graduate School of Life and Environmental Sciences, Kyoto Prefectural University, Hangi-cho, Shimogamo, Sakyo-ku, Kyoto 606-8522, Japan; tsukahara@kpu.ac.jp; 3 Department of Community Perinatal Medicine, Shiga University of Medical Science, Seta Tsukinowa-cho, Otsu, Shiga 520-2192, Japan

**Keywords:** probiotics, heat-treated, non-live bacteria, humoral immunity, sepsis, serum IgG, stool secretory IgA

## Abstract

Probiotic supplementation has been part of the discussion on methods to enhance humoral immunity. Administration of *Bifidobacterium bifidum* OLB6378 (OLB6378) reduced the incidence of late-onset sepsis in infants. In this non-randomized study, we aimed to determine the effect of administration of live OLB6378 on infants’ humoral immunity. Secondly, we tried to elucidate whether similar effects would be observed with administration of non-live OLB6378. Low birth weight (LBW) infants weighing 1500–2500 g were divided into three groups: Group N (no intervention), Group L (administered live OLB6378 concentrate), and Group H (administered non-live OLB6378 concentrate). The interventions were started within 48 h after birth and continued until six months of age. Serum immunoglobulin G (IgG) levels (IgG at one month/IgG at birth) were significantly higher in Group L than in Group N (*p* < 0.01). Group H exhibited significantly higher serum IgG levels (*p* < 0.01) at one month of age and significantly higher intestinal secretory immunoglobulin A (SIgA) levels (*p* < 0.05) at one and two months of age than Group N. No difference was observed in the mortality or morbidity between groups. Thus, OLB6378 administration in LBW infants enhanced humoral immunity, and non-live OLB6378, which is more useful as a food ingredient, showed a more marked effect than the viable bacteria.

## 1. Introduction

Infants are at risk of contracting infectious diseases due to low humoral immunity [1]. Their high risk of early infantile infectious disease can be partially attributed to the insufficient production of serum immunoglobulin G (IgG) and intestinal secretory immunoglobulin A (SIgA) antibodies, both of which are the main components of systemic and mucosal humoral immunity. Infants with a low serum IgG level are at a high risk of developing sepsis and the levels of SIgA in the gastrointestinal system have been shown to affect the incidence of infectious diseases [2,3,4,5]. Thus, enhancing humoral immunity within an appropriate range during the early stages could be an effective strategy to ensure infants’ healthy growth and development without contracting serious infections. In recent years, probiotic supplementation has been at the center of the discussion on effective methods to enhance humoral immunity [6,7]. Several groups have reported cases in which supplementing infants with probiotics increased either serum IgG or intestinal SIgA levels [8,9,10,11]. Thus, if it is possible to enhance infants’ humoral immunity (both serum IgG and intestinal SIgA) with a single probiotic, the efficacy for increasing anti-infective protection would be potentially high.

*Bifidobacterium bifidum* OLB6378 (OLB6378), which belongs to the *Bifidobacterium* genus, could be a strong probiotic candidate that is capable of enhancing infants’ overall humoral immunity. Bifidobacteria, which can inhibit pathogenic microorganism infections by increasing the humoral immunity, are the most predominant bacteria in the gut flora of breastfed infants [12]. OLB6378 is a strain of bacteria that is prevalent in the gastrointestinal tracts of infants and a strain of *B. bifidum* with a high SIgA production-inducing activity [13]. In our previous multicenter study [14], enteral administration of live OLB6378 reduced the incidence of late-onset sepsis in very low birth weight (VLBW) infants, suggesting that live OLB6378 administration could enhance humoral immunity (e.g., an increase in serum IgG).

Similar to live OLB6378, non-live OLB6378 administration may have the potential to enhance anti-infective protection or humoral immunity in infants. Experimental animal studies have suggested a role of non-live probiotics in immunomodulation [15,16]. Although the effects of enteral administration of non-live OLB6378 on serum IgG and intestinal SIgA production have not been studied to date, an in vitro study has shown an increased expression of a polymeric immunoglobulin receptor, linked to SIgA production, by non-live OLB6378 [17]. Since non-live OLB6378 generally have long-term stability [5], they may prove to be a technically easier way of enhancing the infant’s humoral immunity compared to live bacteria. Therefore, we evaluated whether supplementation with live or non-live OLB6378 affected the enhancement of humoral immunity in low birth weight (LBW) infants.

## 2. Materials and Methods 

### 2.1. Ethical Statement

This study was registered in the UMIN Clinical Trial Registry (UMIN000020520) and was conducted according to the Declaration of Helsinki. All procedures were approved by the Institutional Review Board of Shiga University of Medical Science.

### 2.2. Subjects and Study Protocol

The subjects of the study were selected among infants with a LBW (1500–2500 g) admitted to the Shiga University of Medical Science Hospital’s neonatal intensive care unit (NICU) between March 2013 and May 2014 and whose legal guardians signed a proper informed consent for participation in the study within 48 h after birth. The infants whose legal guardians refused to sign the informed consent and infants with congenital malformations unlikely to survive to 6 months were excluded from the study.

We selected the method of determining groups by entry order in order to minimize the chance of errors with administration of study intervention and cross-colonization. The subjects were divided into three groups: Group L (administered live OLB6378 concentrate), Group H (administered non-live OLB6378 concentrate), and Group N (control group) in a 1:1:1 proportion, such that each group had >30 subjects. The sample size was determined based on a previous study on the effect of probiotics [10]. We conducted a non-randomized evaluation and entries were made in the following order: Group H (March 2013–July 2013), Group L (August 2013–November 2013), and Group N (December 2013–May 2014). Multiple-birth infants were all assigned to the same group. To prevent accidental probiotic administration by the legal guardians of the infants following hospital discharge, we assigned multiple-birth parents to the same group. The persons responsible for medical examinations and assessments in this study were blinded to the subjects’ group, and the legal guardians were not informed of the type of the administered trial compound.

### 2.3. Study Intervention

We used live OLB6378 powder (Meiji Co., Ltd., Tokyo, Japan), a lyophilized probiotic powder that contains 0.5 g/g of live OLB6378 concentrate, 0.25 g/g of sucrose, and 0.25 g/g of trehalose. For non-live OLB6378, we dissolved live OLB6378 powder in sterile water with a concentration of 10% and lyophilized them by freeze-drying following a 10 min heat treatment at 80 °C. Heat-treated batches were subjected to a culture-test to ensure that they did not contain any live bacteria.

Group N received no intervention. Group L was administered a mixture of 20 mg of live OLB6378 powder (containing 10 mg of lyophilized live OLB6378 concentrate with >2.5 × 10^9^ live cells) and 480 mg of dextrin (Matsutani Chemical Industry Co., Ltd., Itami, Japan), which is same as the dose used in our previous multicenter study [14]. Group H was administered a mixture of 20 mg of lyophilized non-live OLB6378 powder (containing 10 mg of lyophilized non-live OLB6378 concentrate with >2.5 × 10^9^ non-live cells) and 480 mg of dextrin. Each mixture (500 mg) was diluted in 1 mL of warm water, mixed with either breast milk or infant formula (depending on the extent of lactation in the mothers), and divided into two 250 mg doses. A 250 mg dose was administered in each group twice daily. The interventions were started within 48 h after the infants’ birth and continued until six months of age. Prior to discharge from the NICU, parents of the infants in groups L and H were instructed on how to administer the trial compound to their infants at home, and were requested to perform definite intervention. Furthermore, after discharge from the NICU, compliance with the use of the trial compound was assessed by interviewing the parents every month up to six months of age.

To eliminate the effects of cross-contamination of live OLB6378 within the NICU, infants from Groups H and N whose stool at one month was positive for OLB6378 by the Toshimitsu method [18] were excluded from the data analysis. 

### 2.4. Specimen Collection and Laboratory Methods

Blood and stools of the subjects at birth and at one, two, and six months of age were collected. The collected blood samples were immediately tested at a laboratory in Shiga University of Medical Science Hospital to measure the serum IgG concentrations using the nephelometry method (BM6070, JEOL Ltd., Akishima, Japan). Serum IgG levels were calculated as the ratio of serum IgG concentrations at each month (one, two, and six months) to those at birth. The collected stool samples were immediately frozen at −20 °C. Then, the stool samples were mixed with nine volumes of phosphate-buffered saline and homogenized. The homogenates were centrifuged at 20,000× *g* for 15 min at 4 °C and the supernatant were used for quantification of SIgA. A commercial ELISA kit (Human IgA ELISA Quantitation Set, Bethyl Laboratories, Inc., Montgomery, TX, USA) was used to measure SIgA concentrations in the stool according by the manufacture’s instruction. Stool samples that were too small to be measured (<2.5 mg) were excluded. The technicians who measured the serum IgG and stool SIgA levels were blinded to the subjects’ groups. 

### 2.5. Outcomes

The primary outcome was defined as serum IgG levels and stool SIgA levels. Mortality and morbidity as secondary outcomes from birth to 6 months of age were recorded. Morbidity was defined as the incidence of sepsis and severe digestive symptoms due to OLB6378 requiring treatment. The definition of sepsis was established as a positive test result for pathogenic microorganisms from the cultured blood of infants; appearance of clinical symptoms such as apnea and a general feeling that the newborn is lethargic; or suspected infection based on test findings such as hyperglycemia that blood glucose levels is higher than 200 mg/dL and neutrophil left shift that the immature neutrophils are over 20% of the total neutrophils. The definition of severe digestive symptoms was established as abdominal distension; feeding intolerance; bilious vomiting; diarrhea and possible signs of intolerance to the study intervention. We collected the data on severe digestive symptoms via retrospective chart review.

### 2.6. Statistical Analysis

Non-normally distributed data were subjected to log transformation before analysis. Differences were evaluated by Fisher’s exact test or one-way ANOVA with Dunnett post hoc test using the Bell Curve for Excel (Social Survey Research Information Co., Ltd. Tokyo, Japan). Differences were considered significant at *p* < 0.05.

## 3. Results

### 3.1. Background Characteristics

**Figure 1:**
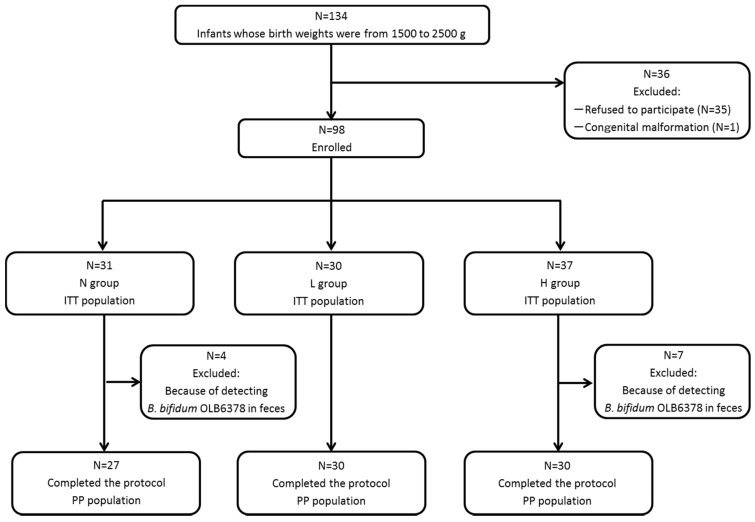
Classification of subjects. N group = control; L group = live OLB6378; H group = non-live OLB6378; ITT population = intention-to-treat population; PP population = per-protocol population.

As shown in Figure 1, 134 infants with a birth weight between 1500 and 2500 g were admitted to the Shiga University of Medical Science Hospital’s NICU, 35 of which were excluded from the study because consent for participation could not be obtained from their legal guardians and one was excluded because of a congenital malformation syndrome. A total of 98 infants (the intent-to-treat population) were included in the study: Group L, 30; Group H, 37; and Group N, 31. OLB6378 was detected in all stool samples from Group L. Seven infants from Group H and four from Group N whose stool samples at one month of age tested positive for OLB6378 by real-time PCR analysis were further excluded.

The final analysis was conducted for 87 infants in total (the per-protocol population): Group L, 30; Group H, 30; and Group N, 27. Table 1 shows that among the three groups, no significant differences were noted with regard to the subjects’ or the intent-to-treat population’s characteristics, such as body weight, gestational age, Apgar score, sex, caesarean section or multiple birth.

### 3.2. Outcomes

**Figure 2:**
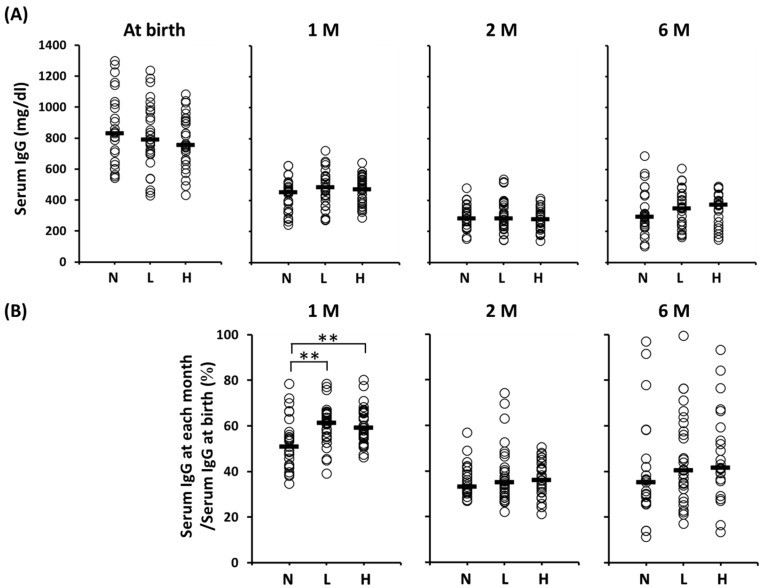
Serum IgG levels of the per-protocol population at birth to six months of age. (**A**) Serum IgG concentration from at birth to six months of age; (**B**) The ratio of serum IgG levels at each month after birth to serum IgG levels at birth. Horizontal lines represent median values. ** Statistically different based on a Dunnett test (*p* < 0.01). Serum IgG levels at one month of age: N vs. L, *p* = 0.0023; N vs. H, *p* = 0.0026. 1 M = one month of age; 2 M = two months of age; 6 M = six months of age; N = no intervention control; L = live OLB6378; H = non-live OLB6378.

Figure 2 shows the serum IgG concentrations at each time point and the ratio of the serum IgG levels (IgG at each month/IgG at birth). No significant difference in the serum IgG concentrations among the groups throughout the duration of the study was observed. However, the concentrations decreased progressively from the time of birth throughout the study period. The serum IgG levels (%, median (range)) in Groups L (61 (39–79)) and H (59 (46–80)) at one month of age were significantly higher than those in Group N (51 (35–78)), although no significant differences among these groups at two or six months of age were found.

**Figure 3:**
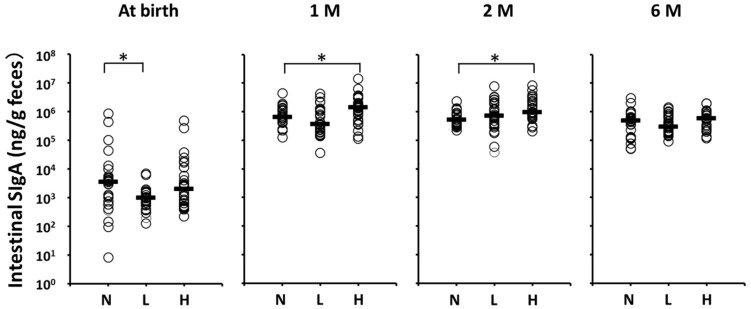
Fecal SIgA levels of the per-protocol population at each month after birth. Horizontal lines represent median values. * Statistically different based on a Dunnett test (*p* < 0.05). N vs. L at birth, *p* = 0.024; N vs. H at one month of age, *p* = 0.031; N vs. H at two months of age, *p* = 0.0055. 1 M = one month of age; 2 M = two months of age; 6 M = six months of age; N = no intervention control; L = live OLB6378; H = non-live OLB6378.

Figure 3 shows the stool SIgA levels of the per-protocol population. Although postnatal stool SIgA levels (μg/mL, median (range)) were significantly lower in Group L (1.0 (0.1–6.7)) than in Group N (3.5 (0.008–830.4)), no significant differences between Groups L and N at one, two, and six months of age were noted. In contrast, postnatal stool SIgA levels at one and two months of age were significantly higher in Group H than in Group N (1399.0 (114.1–14110.0) vs. 642.6 (128.1–4432.0) at one month, 966.1 (207.3–8487.0) vs. 519.4 (223.8–2350.0) at two months, respectively).

There was no case of sepsis or severe digestive symptoms in the three groups (no probiotic sepsis in group L). No significant difference was observed in the mortality or morbidity, i.e., sepsis or severe digestive symptoms requiring treatment, among the groups (Table 1). In Groups L and H, no particular side effects or subject withdrawals from the study were noted.

## 4. Discussion

In this non-randomized study, we established that early postnatal OLB6378 administration potentially increases anti-infective protection by enhancing humoral immunity through a mechanism that increases both serum IgG and intestinal SIgA levels. Of interest, non-live OLB6378 exhibited this effect more markedly than live OLB6378. To the best of our knowledge, this is the first study to establish that non-live probiotics simultaneously increased both serum IgG and intestinal SIgA levels in infants.

Initially, we found that early postnatal OLB6378 administration results in an early increase in the production of serum IgG, which is the main contributor to the attenuation of infection in early infancy. We established that serum IgG levels (IgG at one month/IgG at birth) in infants supplemented with live OLB6378 were significantly higher compared to the control group. This effect was observed not only in live OLB6378 administration but also in non-live OLB6378. The serum IgG transfer from mother to fetus occurs mainly in the third trimester [19]. Maternal IgG is metabolized and serum IgG gradually decreases, reaching the lowest point at the third to sixth month of the infant’s life. Thereafter, serum IgG levels rise due to an increase in infants’ endogenous production of IgG [20,21]. In this study, the period during which an increase in serum IgG levels due to OLB6378 administration was observed corresponded to the period when LBW infants were susceptible to late-onset sepsis (i.e., at one month of age). Therefore, in relation to the mechanism that reduced the incidence of late-onset sepsis in VLBW infants in our previous multicenter study [14], our findings strongly suggest a possibility that OLB6378 administration accelerated the endogenous production of serum IgG, which compensated for a decreased supply of maternal IgG, thus maintaining high levels of serum.

Early postnatal OLB6378 administration also resulted in an early increase in the intestinal SIgA concentration, which is important for the prevention of infections in infants. We observed a significant increase in intestinal SIgA levels at one and two months of age in infants with early postnatal non-live OLB6378 administration. This period corresponded to the period in which the infants’ endogenous production of intestinal SIgA increases [22,23,24,25]. This suggests that OLB6378 administration can increase infants’ endogenous intestinal SIgA production. Infants’ intestinal SIgA is supplied through passive immunity [26]. However, some infants cannot be breastfed for various reasons. In these infants, improving the anti-infective protection is suggested by increasing the rate of the endogenous production of intestinal SIgA through early postnatal OLB6378 administration, thereby compensating for the insufficient passive immunity.

Interestingly, non-live bacteria increased intestinal SIgA levels more markedly than the live bacteria in this study. This result is consistent with the results of the study in which oral administration of non-live *Lactobacillus plantarum* in mice resulted in a greater intestinal SIgA production compared with that of live bacteria [16]. Generally, probiotic action is not only manifested through the improvements in gut microbiota but is also exhibited through the stimulation of the host’s immune responses by the components of bacteria [27]. Such stimulation of immune responses occurs through Toll-like receptors expressed on the membranes of leukocytes. It has been reported that some probiotics increased the amount of IgA and Toll-like receptor signaling is critical for this effect [28]. Although the mechanism that increases intestinal SIgA production due to OLB6378 remains unclear, further findings may be obtained from future studies on the possibility that the changes in the structure or components of OLB6378 by heat-treating boost immunostimulation through Toll-like receptors. However, some groups have reported that supplementing full-term infants with either *Lactobacillus rhamnosus* GG or a combination of *Bifidobacterium longum* BB536 and *L. rhamnosus* LPR increased serum IgG levels, and supplementing pre-term or full-term infants with *Bifidobacterium lactis* Bb12 increased fecal SIgA levels [8,9,10,11]. These studies indicated that a single probiotic can elevate either serum IgG or intestinal SIgA in infants. This is the first human study in LBW infants to demonstrate an increase both serum IgG and SIgA following administration of non-live OLB6378.

We found that non-live OLB6378 administrations are safe and well tolerated in infants, as were the live bacteria. In our previous multicenter study, live OLB6378 administration in VLBW infants showed no increase in the incidence of adverse effects [14]. In this study, we, for the first time, established that no adverse effects were observed with non-live OLB6378 administration in LBW (1500–2500 g) infants. Nevertheless, some studies have shown cases in which sepsis (also called probiotic sepsis) developed in patients following the administration of live bacteria, which is one of the possible adverse effects of probiotics [29]. In this respect, it can be stated that administering non-live OLB6378 in infants has a high safety profile.

This study has several limitations. First, the conditions for subject selection differed from those in our previous multicenter study [14]. Specifically, in this study, we targeted LBW infants weighing between 1500 and 2500 g. Since this is the first clinical trial with non-live OLB6378, the birth weight of the range of subjects was set higher than that in our previous study for safety concerns. For the same reason, we conducted this study in a single facility, unlike our previous study which was conducted in multiple facilities. Thus, although it was speculated that OLB6378 administration is one of the factors that reduced the incidence of late-onset sepsis by enhancing infants’ humoral immunity, the findings of this study may not be applicable to VLBW infants. Second, this study is not a randomized blind clinical trial, but a comparative single blind trial. We selected the method of determining groups by entry order in order to minimize the chance of errors with the administration of the study intervention and cross-colonization, because Hickey et al. demonstrated the cross-colonization of infants with probiotic organisms in the neonatal unit [30]. In addition, because the subjects were infants, this study retained the condition of a blind trial. Although placebo was not employed in this study, a control group, which received no trial compounds and was under the same conditions as the non-control groups, was used. Though entries for each group were from different periods, no changes were observed in the environment in relation to possible cross-colonization, e.g., a nursing-to-patient ratio that could affect the outcome. Therefore, this single blind comparative study, in our opinion, yielded highly objective results. Finally, in this study, the mode of feeding was different among the subjects. The mode of feeding had a significant impact on fecal SIgA levels in full-term infants [22]. However, in this study, an analysis of the relationship between fecal SIgA levels and the mode of feeding (obtained from the records of the formula intake per body weight) in the control group, at one and two months, demonstrated that the impact of the mode of feeding on fecal SIgA levels was low (Figure S1). The reason for this is unknown; however, it might be related to the fact that the subjects of our study were LBW infants but not full-term infants.

Here, we observed that early postnatal OLB6378 administration increases both serum IgG and intestinal SIgA levels. This enhancement of humoral immunity may increase anti-infective protection in infants. Because serum IgG and intestinal SIgA play a central role in systemic and intestinal protection against infections, early postnatal OLB6378 administration, which, as we established, may reduce the incidence of late-onset sepsis [14], might also exhibit protective effects against other types of infectious diseases. The impact of probiotics on late-onset sepsis in VLBW infants has recently been discussed in a large meta-analysis [31]. Non-live OLB6378 administration could be safe and beneficial to VLBW infants. Moreover, the increase in humoral immunity through non-live OLB6378 administration to full-term infants might be useful in the prevention of infantile allergies [32]. Further studies on the effects of non-live OLB6378 administration on not only VLBW infants but also on full-term infants in the early postnatal period are warranted.

## 5. Conclusions 

We conclude that live OLB6378 can enhance humoral immunity in LBW infants and that this effect is more marked following the administration of non-live OLB6378.

## Figures and Tables

**Table 1:** Characteristics and outcomes of the intent-to-treat population.

	N Group *n* = 31	L Group *n* = 30	H Group *n* = 37	Difference
**Characteristics**				
Gestational age, week ^a^	35.4 ± 1.8	35.1 ± 1.4	34.6 ± 1.7	N.S. †
Body weight at birth, g ^a^	2027 ± 264	2077 ± 208	1957 ± 244	N.S. †
Body weight at 6 months, g ^a^	6915 ± 729	6889 ± 662	7015 ± 736	N.S. †
Apgar score at 1 min ≤ 3 ^b^	0 (0)	0 (0)	0 (0)	N.S. ‡
Apgar score at 5 min ≥ 7 ^b^	31 (100)	30 (100)	37 (100)	N.S. ‡
Male sex ^b^	14 (45)	16 (53)	18 (49)	N.S. ‡
Caesarean section ^b^	25 (80)	24 (80)	31 (80)	N.S. ‡
Multiple pregnancy ^b^	16 (51)	16 (53)	20 (54)	N.S. ‡
**Outcomes**				
Mortality ^b^	0 (0)	0 (0)	0 (0)	N.S. ‡
Sepsis ^b^	0 (0)	0 (0)	0 (0)	N.S. ‡
Severe digestive symptom ^b^	0 (0)	0 (0)	0 (0)	N.S. ‡

N group = control; L group = live OLB6378; H group = non-live OLB6378; ^a^ Mean ± standard deviation, ^b^ Number (%), † One-way ANOVA, ‡ Fisher’s exact test.

## Supplementary Materials

The following are available online at http://www.mdpi.com/2072-6643/9/3/195/s1, Figure S1: Relationship between the fecal SIgA levels and formula intake.
