# Supplementary material for: Bifidobacterium bifidum OLB6378 Simultaneously Enhances Systemic and Mucosal Humoral Immunity in Low Birth Weight Infants: A Non-Randomized Study
Source: Nutrients. 2017 Feb 26;9(3):195. doi: 10.3390/nu9030195 (PMC5372858; doi:10.3390/nu9030195)
Supplement: Supplementary file 1 [file nutrients-09-00195-s001.pdf]

Bifidobacterium bifidum OLB6378 Simultaneously Enhances Systemic and Mucosal Humoral Immunity in Low Birth Weight Infants: A Non-Randomized Study

Katsunori Tanaka, Takamitsu Tsukahara, Takahide Yanagi, Sayuri Nakahara, Ouki Furukawa, Hidemi Tsutsui and Shigeki Koshida

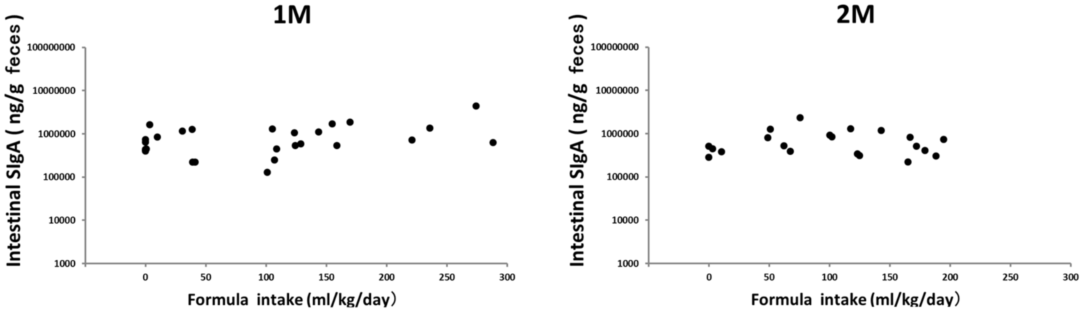

Figure S1. Relationship between the fecal SIgA levels and the formula intake of group N. The graph demonstrates the independence of fecal SIgA levels versus formula intake, and no significant correlation between the fecal SIgA levels and the formula intake was observed. Data were analyzed using the Spearman's rank correlation coefficient ($p < 0.05$).
